# Developing a typology of network alteration strategies for implementation: a scoping review and iterative synthesis

**DOI:** 10.1186/s13012-023-01266-3

**Published:** 2023-04-06

**Authors:** Alicia C. Bunger, Reza Yousefi-Nooraie, Keith Warren, Qiuchang Cao, Porooshat Dadgostar, Tatiana E. Bustos

**Affiliations:** 1 College of Social Work, The Ohio State University, Columbus, OH USA; 2 Department of Public Health Sciences, University of Rochester, Rochester, NY USA; 3 Pepper Institute On Aging and Public Policy & Claude Pepper Center, Florida State University, Tallahassee, FL USA; 4 Department of Psychology, Michigan State University, East Lansing, MI USA

**Keywords:** Social networks, Network interventions, Implementation strategies, Specification, Typology

## Abstract

**Background:**

Social networks transmit knowledge, influence, and resources. These relationships among patients, professionals, and organizations can shape how innovations are disseminated, adopted, implemented, and sustained. Network alteration interventions—interventions that change or rewire social networks—have the potential to be used as implementation strategies. Yet, the types, mechanisms, and effectiveness of these interventions for implementation are unclear. This scoping review and iterative synthesis identified and described network alteration strategies that could be tested for implementation.

**Methods:**

We used forward and backward citation tracking of influential articles on network interventions, bibliometric searches, and hand searches of peer-reviewed social network journals. At least two team members screened article titles/abstracts to identify studies that met inclusion criteria: empirical studies of an intervention, the intervention was designed to alter some element of a social network, and changes in social network metrics were measured at two or more time points. During full-text reviews, information about the network interventions, actors, ties, and main findings was extracted. Reporting was informed by the Preferred Reporting Items for Systematic Reviews and Meta-Analysis (PRISMA). To develop our typology, we synthesized these results using an iterative team-based and consensus-building process.

**Results:**

Fifty-three articles met the inclusion criteria. The interventions described were conducted in healthcare systems or behavioral health systems (34%), communities (26.4%), and schools (22.6%). The majority included records describing interventions designed to alter social support, information-sharing, or friendship networks (65%) among individual actors (84.9%), or to increase ties. Eight strategies emerged. Three strategies targeted the general context: (1) change the environment, (2) create groups, and (3) change the composition. Four strategies targeted individual actors: change (4) motivations, (5) skills for networking, (6) knowledge of one’s social network, and (7) prominence/roles. One strategy (8) targeted specific ties within the network (targeting a particular pair-wise relationship or changing the nature of an existing tie).

**Conclusion:**

The network alteration strategies in this typology provide further operational specificity for how implementation strategies target relationships. Advancing these strategies will require greater theoretical specification, the development of strategies that target professionals and organizations, and studies that examine the impact on implementation outcomes.

**Supplementary Information:**

The online version contains supplementary material available at 10.1186/s13012-023-01266-3.

Contributions to the literature

- Many implementation strategies focus on building social networks but lack specificity.
- Network alteration interventions purposefully build or rewire social networks. These interventions have the potential to be used as implementation strategies.
- We identified 8 network alteration strategies that could be used for implementation. Three strategies targeted the general context: (1) change the environment, (2) create groups, and (3) change the composition. Four strategies targeted individual actors: change (4) motivations, (5) skills for networking, (6) knowledge of one’s social network, and (7) prominence/roles. One strategy (8) targeted specific ties.
- We offer the next steps for testing these strategies on implementation outcomes.

## Introduction

Knowledge, influence, and resources spread through the social networks of patients, professionals, and organizations. Social networks enhance or impede innovation adoption, implementation, and sustainment [1–3]. Unsurprisingly, many implementation strategies include relational interactions and network-building components [4]. For instance, network weaving, coalition building, and developing resource-sharing agreements build relationships among implementation actors to support engagement, knowledge sharing, and resource exchange [5, 6]. These strategies could be considered network alteration interventions, which are deliberate efforts to change networks by adding or deleting actors or relationships to form, strengthen, dissolve, or otherwise change social networks [7]. Despite the existence of established recommendations for specifying the operational details of implementation strategies, approaches for altering networks during implementation are ambiguous. What remains unclear are the specific network alteration strategy types, determinants of their success, an understanding of their mechanisms of action, and evidence of effectiveness. These gaps limit understanding and ability to implement innovations within complex healthcare and social contexts. The purpose of this scoping review and synthesis is to identify and provide a classification of network alteration strategies that have been tested and could be used to further specify implementation strategies.

### Social Networks and Implementation

Networks are a set of actors (nodes) connected through their relationships [8]. Actors might represent individuals, groups, or organizations. Relationships among actors might be based on familial or friendship ties, communication, collaboration, financial exchanges, or any other types of interaction. These relationships serve as conduits for information and other resources; therefore, analysis of the structural features of networks (social network analysis) is often used to draw insights about communication and information-sharing processes, the dynamics of resource flow, and the role of networks in individual behavior change and outcomes [9].

Networks among organizations, clinicians, and patients feature prominently in implementation science. Drawing from Roger’s classic diffusion of innovation theory, information, resources, and influence are diffused or communicated through social relationships [10]. In the external environment (or outer setting of health and human service delivery), external collaborations and connections among organizations introduce new information, influence, innovations, and resources that can lead to adoption decisions and strong implementation [11–14]. Within the internal organizational environment, social networks among professionals can shape attitudes, beliefs, learning, and implementation [12, 15–19]. Regardless of whether the focus is on external or internal networks, these social relationships reflect critical transactional processes that can lead to transformations necessary for implementation [20].

### Social networks change

Social networks are not static—relationships change naturally over time as actors build new, strengthen existing, or dissolve old ties. Indeed, several studies have observed this natural network evolution among clinicians and organizations during implementation and quality improvement initiatives demonstrating that the network context changes [21, 22]. Interpersonal and interorganizational networks evolve based on actors’ needs, choices, and existing relationships [23–27]. For instance, actors tend to form direct relationships with those who are similar, accessible, or influential to them, tend to reciprocate connections, and connect with those with whom their partners are connected (forming closed triads or clusters [25, 28]. These natural tendencies to form and strengthen connections are considered *endogenous* network effects.

However, networks also change because of *exogenous* or externally imposed effects. While some exogenous effects could be triggered by an external shock (e.g., natural disaster, funding fluctuations), others can be planned and thoughtful network interventions. These deliberate efforts to change networks were defined as *network alteration interventions* in an influential article by Valente in 2012 [7] and have the potential for organized dissemination and implementation efforts [29]. Valente (2012) describes alteration interventions as a type of network intervention that (1) introduces or removes actors, (2) introduces or removes ties, or (3) modifies existing relationships. These alterations change network structures and theoretically produce a desired behavior or outcome.

As Valente [7] describes, network alteration interventions can target interpersonal social networks by introducing lay health advisors, removing individuals from sexual contact networks, or introducing a “buddy system.” Interventions might also change interorganizational networks, including large group interaction methods advanced in the trans-organizational development field (interventions intended to build and strengthen relationships across multiple organizations) [30]. Search conferences, for example, are large group participatory planning approaches that bring multiple organizational representatives together; the intention is to change networks by fostering new partnerships that can be used for collaborative strategic planning, governance, and problem-solving [31].

### Implementation strategies can alter social networks

Although not labeled explicitly as network alteration interventions, many existing implementation strategies change individual or organizational relationships [5, 6]. In fact, the Expert Recommendations for Implementing Change (ERIC) strategy taxonomy includes 17 strategies within the broader domain of strategies for building stakeholder interrelationships [6, 32]. For example, learning collaboratives bring together clinician teams from multiple organizations to promote shared learning; these strategies alter social networks among clinicians by building ties with local practice experts, strengthening communication ties within agency teams, and dissolving advice-based ties with external colleagues who are not using the focal evidence-based intervention [33–35]. Similarly, strategies like building a coalition, sharing local knowledge, and creating new clinician teams have the potential to foster new social ties among actors. In addition, introducing data experts and building relationships with academic partners theoretically introduce new actors into networks. Indeed, a recent review of ERIC taxonomy implementation strategies noted that half were relational in some way [4].

Despite the common use of interventions and implementation strategies that change social networks, they have received limited conceptual and empirical attention [30, 36, 37]. One notable exception is Gesell and colleagues’ [36] Social Networks Diagnostic tool which was developed to help group interventionists in their study build social networks among participants based on measured network properties. For instance, to build connections among participants who are isolates (not having any direct ties to other actors) or have low degree scores (the number of ties with other actors), interventionists deliberately pair them with others in the network. In situations where there is low network density (the overall connectedness among group members), interventionists are encouraged to lead community-building activities to build and strengthen relationships. Gesell and colleagues found that these approaches changed social networks among participants over time but called for deeper exploration of the mechanisms of action that lead to changes in the network, and, ultimately, practice or behavior change among actors [38]. However, a recent review of studies that applied social network analysis in health found that studies of network alteration interventions remain rare [37]. As a result, specific methods for altering social networks, resulting network changes, and effects on implementation, service delivery, and client outcomes remain unclear.

Because network alteration interventions have the potential to inform and further specify implementation strategies, the purpose of this study is to develop a typology of network alteration strategies. This typology is intended to lay the foundation for further specifying implementation strategies, understanding their mechanisms of action, and testing their effectiveness.

## Methods

### General design

To identify network alteration interventions that could be used in implementation, we developed a scoping review protocol consistent with Arksey and O’Malley’s framework [39] and the Preferred Reporting Items for Systematic Reviews and Meta-Analyses (PRISMA) guideline [40, 41]. These procedures were intended to identify interventions designed to alter network structures in different contexts, characterize key features, and further build on Valente’s (2012) concept of network alteration interventions. To develop our typology, we synthesized these results using an iterative and team-based consensus-building process using rounds of inductive and deductive coding. This protocol has not been previously registered.

### Review questions

1. Our review addressed:
2. What types of networks (settings, actors, ties) have been examined in studies of network alteration interventions?
3. What structural features of the network were targeted by network alteration intervention strategies in the literature?
4. What types of network alteration intervention strategies have been reported in the literature?

### Identifying relevant literature

**Fig. 1:**
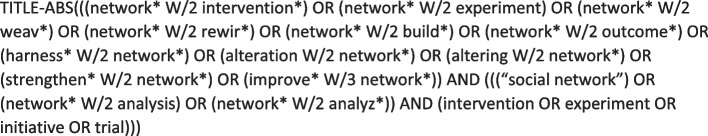
Search terms

We searched for relevant literature in four ways. We began with three influential articles that are well-known and cited for defining network interventions [7], describing the role of networks in implementation (1), and network interventions in health promotion and implementation [42]. First, we conducted backward citation tracking by identifying all articles cited in the three influential articles [1, 7, 42]. Second, we conducted forward citation tracking and identified all works that cited the three influential papers since their publication (until October 2019). Third, we complemented this search approach with a bibliometric search to identify network interventions not included in the backward/forward citation tracking. To capture a broad range of network intervention contributions beyond the health and social care contexts that were the focus of the three influential papers, we searched for articles that reported on a range of network intervention terms and synonyms (Fig. 1) in the title or abstract in two multidisciplinary scholarly databases (Web of Science and Scopus) published until October 2019. Fourth, we scanned the table of contents of two recognized network analysis journals *Social Networks* and *CONNECTIONS* for intervention-relevant titles. Our citation tracking and the bibliometric search yielded 3390 unduplicated articles which were managed in Mendeley and Covidence.

### Article selection (screening)

Articles were included if they were: (a) empirical studies of interventions designed to change (alter) some element of a social network, and (b) measured changes in networks, or some element of the network structure (egocentric or socio-centric) at two or more time points. Articles were excluded if they were (a) not published in peer-reviewed journals (including technical reports, and gray literature), (b) theoretical or conceptual papers that did not include empirical assessments, (c) cross-sectional or post-test only designs, (d) analyzing networks of non-social entities (e.g., neural, IT, or genetic networks), (e) not available in full-text English, or (f) about interventions or events that were not designed to change social networks (where network change might have been an unexpected byproduct of the intervention, like a natural disaster). Given the diversity of the articles generated, these inclusion and exclusion criteria were developed, applied, and refined iteratively by our team, comprised of researchers and trainees with expertise in social network analysis.

**Fig. 2:**
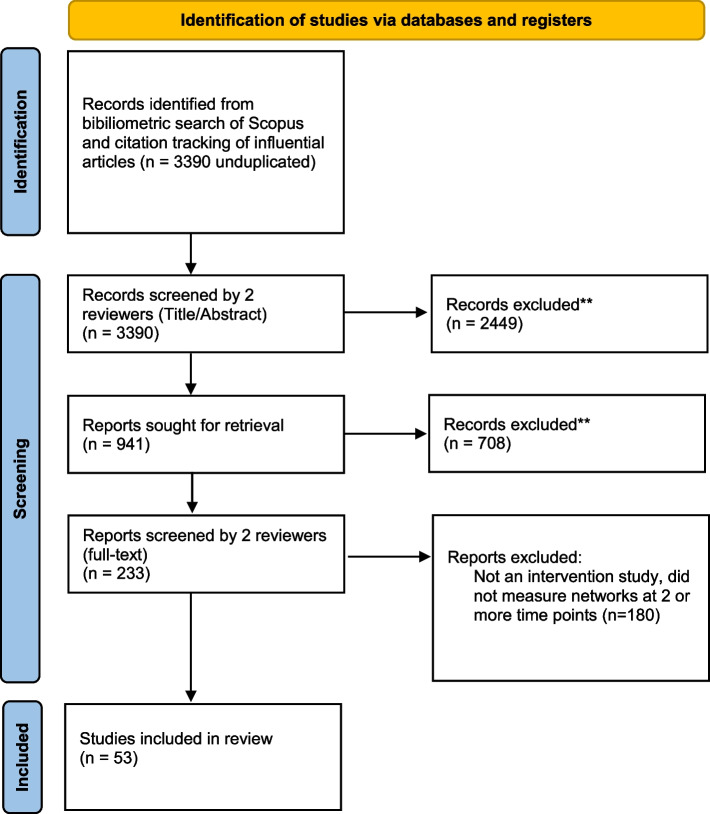
PRISMA diagram

To select articles, each title and abstract was first reviewed against the inclusion/exclusion criteria by at least two members of the team. Most articles excluded at this stage were from the gray literature, non-empirical, or focused on non-social networks. The initial screening generated 941 articles. To enhance the reliability of our search process, we added a second review to ensure articles met the inclusion criteria; two team members scanned the full-text article for information about the intervention and network metrics assessed. In cases where screeners were unsure or disagreed with one another, a third team member made final decisions. This process excluded 822 articles; most were excluded because there was no specific intervention described, or because social networks were not measured at multiple time points. This yielded 233 articles. All articles were double-coded, with at least two reviewers assigned to each article to conduct an in-depth full-text read and confirm the review’s inclusion criteria. At this stage, we retained a final set of 53 records (Fig. 2—PRISMA Diagram) [43].

### Data charting

We developed an initial data charting form to extract key intervention features aligned with our guiding questions (Table 1). This included basic categorical information about each study and open text descriptions to capture overall study approaches. We drew from our experience in network analysis on individual and organizational system levels (e.g., [22, 44–47] to specify and categorize common types of nodes (e.g., children, organizations), ties (e.g., information-sharing, friendship, advice-seeking), metrics (e.g., density, centralization, clustering), and other features of each study. This form was pilot tested by the team—each team member used the form to extract information on a small subset of articles. We then reviewed our experiences and results and refined the form iteratively to clarify concepts, definitions, and their application during monthly team meetings.

**Table 1:** Initial data charting fields

Field	Definition/instructions
Coder initial	Initials of the team member
Article ID	Internal identification number for article
Article first author	Last name of the article’s first author
Year	Year article was published
Article title	Full title of the article
Journal	Name of Journal
Type of nodes/actors	The actors/nodes targeted by the intervention
Types of ties/relationships	The types of ties or relationships that were examined among the nodes
Setting	The type of setting in which the study/intervention was conducted
Intervention facilitator	Whether the intervention (1) has an external facilitator (designated individual managing the intervention by coaching participants, brokering relationships, or other approach) or (2) relies on natural networking tendencies to drive change
Intervention name	Name of the intervention (if noted)
Intervention descriptions^a^	One sentence summary of the intervention strategy/approach
Intervention goals^a^	Stated goal, objective, outcome, or purpose of the intervention
Intended network changes^a^	The network features/measures expected to change with the intervention
Actual network change	One sentence description of whether and how the network changed during the intervention

^a^Indicates fields that we focused on to develop our typology

### Collating, summarizing, and synthesizing results

To examine study features (Question 1), we used descriptive statistics (e.g., frequency analysis, cross-tabs). To understand the types of network alteration strategies and outcomes (Questions 2 and 3), we analyzed/synthesized the open-text data extracted during data charting using content analysis. All team members reviewed open-text responses about the intervention description, and intended/actual network changes observed, and developed an initial list of themes. Together, the team discussed and reached consensus on two categories of relevant themes, codes, and definitions: (1) features of the network structure that were targeted during the intervention (e.g., quantity or quality of ties, level of the network) and (2) network alteration strategies. We pilot-tested codes on a subset of three studies (the first three studies in our review) and refined codes based on our experiences (Additional file 1). We expected that the codes would need to be refined as we continued to apply them given the heterogeneity of the interventions. Team members paired up to re-apply this codebook to the included articles, ensuring that all articles were double coded. In cases of disagreement, the article was discussed by the full team to reach consensus. As the codes were refined, teams returned to recode the articles. Information about these interventions was synthesized during our iterative team processes into a typology of network alteration strategies. We drew from Proctor et al.’s) [48] implementation strategy specification guidelines to name the strategy, defined it (general description of the strategy and processes that are enacted), and specified its conceptual target (facets of the network to be impacted.

## Results

### Types of networks targeted by alteration interventions

Of the 53 studies included, 77% were published after Valente’s 2012 network intervention article [7], and the majority were published in health (41.5%) or behavioral health/social science (32.1%) journals. The majority of studies (84.9%) described interventions that altered networks of individuals, while fewer altered networks among organizations, teams/groups/classes, or coalitions (25%) or multi-level networks (10%) (with some overlap across categories). In terms of the type of ties, about two-thirds of the articles described interventions that altered ties that yielded social support (32%), discussion/information-sharing (22.6%), or friendship (11.3%). A substantial percentage of studies focused on collaboration ties (28.3%). Fewer studies focused on influence networks such as those based on advice-sharing (15.1%), or prestige (13.2%). Interventions were conducted in a variety of settings, including health/behavioral healthcare systems (34%), communities (26.4%), and schools (22.6%). Most (73.5%) described using a designated facilitator (e.g., internal leader or volunteer, external staff, or lead organizations) to lead the strategy (Table 2).

**Table 2:** Features of Network Intervention Articles (*n* = 53 records)

Feature	*n*	%
*Journal discipline*		
Health	22	41.5%
Behavioral health/social science	17	32.1%
Engineering/systems	4	7.5%
Education	3	5.7%
Mathematics	3	5.7%
Environmental/development	3	5.7%
Multidisciplinary-open	1	1.9%
*Types of nodes*^a^		
Individuals	45	84.9
Organizations	9	17.0
Teams/groups/classes	3	5.7
Coalitions	1	1.9
*Settings*		
Health/behavioral health	18	34.0
Community	14	26.4
School	12	22.6
Business Firm	3	5.7%
Research	2	3.8
Online	1	1.9
*Tie type*^a^		
Social interactions/social support (emotional support)	17	32.0%
Collaboration (working together)	15	28.3%
Discussion/communication/information (general information)	12	22.6%
Advice/expertise (seeking specialized input, mentorship)	8	15.1%
Influence/prestige/important people	7	13.2%
Friendship (close interpersonal relationship)	6	11.3%
Economic or material resources (e.g., money, goods)	2	3.8%
*Facilitator*	39	73.5%

^a^Categories are not mutually exclusive therefore total more than 100%

### Level of the network and intended outcomes targeted

The majority of articles described network alteration strategies for changing the number/quantity of ties (e.g., network density or actor degree). Fewer (32%) intended to change the qualities of ties, like the frequency or intensity of interactions, or the content of the resources shared in the relationship (e.g., changing a message shared among actors). The majority (75%) targeted individual actor-level changes and examined individuals’ degree or centrality. Nearly half (45.3%) of the studies described interventions designed to alter whole-network-level structures and monitored changes in network-level properties (e.g., density, centralization) (Table 3).

**Table 3:** Network alteration targets (*n* = 53 records)

	*n*	%
*Intended goals*^a^		
Change tie quantity	47	88.7%
Change tie quality	17	32.0%
*Level of network targeted*^a^		
Whole network	24	45.3%
Sub-groups	12	22.6%
Dyads	10	18.9%
Triads	6	11.3%
Individual	40	75.5%
Network boundary	9	17.0%

^a^Categories are not mutually exclusive therefore total more than 100%

### Types of network alteration strategies

Across the 53 articles, eight types of strategies emerged for altering networks. These strategies targeted many facets of the network including the general context for networking (changing the environment, creating groups, and changing the composition, actors’ motivations, skills, knowledge of the network, and prominence), and specific ties (Table 4). Notably, most studies (77.4%) described interventions that used more than one strategy; on average, the interventions used 2.4 strategies (SD = 1.15) and this number ranged from one to five.

**Table 4:** Network alteration strategies (*n* = 53 records)

Name	Definition	Target	*n*	Citations
Create groups	To bring network actors together and align their activities around shared identity or goals in the form of groups. The group could be formed to: 1) provide ‘collective support’ for group participants 2) Provide support for a focal actor 3) Create community around a shared identity (professional identity) 4) Carrying out collective efforts (planning, decision making) 5) Promote shared learning (group training)	Context	30	[30, 36, 38, 49–75]
Change the environment	Modifications or shifts in actors’ environment that lead them to alter their relationships to adapt. These environmental changes can be outside the network boundary: 1) Changing Resources 2) New Opportunities or Events Or within the network: 3) Changing network culture or norms	Context	18	[30, 50–53, 59, 61, 64, 72, 76–84]
Change the composition	Changing the composition of the network by adding and/or removing actors	Context	13	[51, 54, 59–61, 65, 76, 79, 82, 85–88]
Change actors’ networking skills	Changes or improves actors’ skills for connecting or working with others in the network. These strategies can be: 1) Group interventions focused on altering skills for dyadic interactions 2) One-on-one interventions focused on altering skills for dyadic interactions 3) Group interventions on general networking skills 4) One-on-one interventions on general networking skills	Actors	19	[50, 51, 54, 56, 58, 62, 64, 71, 73, 75–77, 85, 86, 88–92]
Change actor awareness and/or knowledge of the network	Builds or changes actors’ knowledge and/or awareness of other actors in the network, and/or their connections to them through: 1) Personal mapping to identify those in an actor’s network 2) Inventories/Directories 3) Labeling specific actors 4)“Get to know you” sessions	Actors	13	[62, 70, 74, 75, 78, 83, 85, 88, 90, 92–95]
Change actor prominence	Changing the centrality (e.g. popularity, mediation, etc.) of some actors (e.g. champions, study participants, or actors with certain characteristics), in relation to others by: 1) Increasing actors’ knowledge or skill through training 2) Decentralizing actors’ with negative behaviors through reinforcements 3) Identifying/labeling actors with expertise as ambassadors or leaders	Actors	9	[22, 62, 78, 84, 90, 91, 95–97]
Change actor motivations to connect	Changing an actors’ motivation to interact with others in the network by: 1) Educating actors’ about benefits/consequences of interactions 2) Incentivizing interaction	Actors	12	[51, 75, 78, 82, 83, 85, 90–93, 95, 96]
Change specific ties	Targeting specific types of relationships, or specific ties between selected actors for formation, strengthening, or dissolution by: 1) Strategically selecting actors to form a group or pair 2) Introduce incentives to form a specific type of relationship 3) Train actors to dissolve a specific relationship	Ties	14	[36, 38, 54, 57, 59, 61, 64, 66, 68, 78, 88, 91, 93, 96]

#### Create groups

Thirty articles (56.6%) described interventions that altered networks by bringing actors together and aligning their activities around a shared identity, interests, or goal [30, 36, 38, 49–75]. These groups were created to foster connections among actors for a variety of purposes, such as: providing social support for group members (e.g., support groups) or a focal actor (e.g., children with developmental disabilities), engaging in collective efforts (e.g., community planning), creating a local community around a shared identity (e.g., professional groups), or facilitating shared learning (e.g., group training). Most of these interventions aimed to increase the connectivity among network actors (*n* = 27, 90%), and fewer sought to change the quality of ties among group members (*n* = 10; 33%).

#### Change the environment

Sixteen articles (30.2%) described interventions that altered networks by changing the environment in ways that led/motivated actors to shift their relationships to adapt [30, 50–53, 59, 61, 64, 72, 76–84]. Environmental change strategies targeted the environment that embeds the network by changing resources (e.g., funding criteria, providing new services), or creating new opportunities to interact (e.g., changes to the physical environment, external shocks, creating an online platform). Some also changed the internal environment (e.g., changes in group culture and norms). Nearly all of these environmental changes were intended to change the number of actors’ connections, and by extension, the connectivity of the whole network (*n* = 17, 94.4%) although several were also designed to improve the quality of ties (*n* = 8, 44.4%).

#### Change the composition

Thirteen articles (24.5%) described interventions that altered networks by changing their composition [51, 54, 59–61, 65, 76, 79, 82, 85–88]. All interventions changed the composition by introducing new actors, including designated counselors, mentors, or peers expected to exert a positive influence on existing network members. Only one article described how a new actor was introduced into a new network for their own benefit. All interventions that changed composition were intended to affect the quantity of ties among actors (*n* = 13, 100%), although 6 studies (*n* = 46%) described how these interventions might also affect tie quality. No interventions removed actors.

#### Change actors’ skills

Nineteen articles (35.8%) described interventions that altered networks by changing actors’ skills for connecting or working with others in the networks [50, 51, 54, 56, 58, 62, 64, 71, 73, 75–77, 85, 86, 88–92]. Most often, strategies were delivered in group settings like workshops (*n* = 17) although some used one-on-one formats (*n* = 3). The skills targeted were tailored to the needs of the individual actors (e.g., children with autism spectrum disorders) and focused on specific types of dyadic relationships (e.g., building reciprocity in mentoring relationships, conflict resolution, parenting) or on networking skills more generally (e.g., engagement, outreach, collaboration, skills for increasing social influence). Several used a combination of actor skill strategies. These strategies aimed to change the quantity (*n* = 15, 78.9%) and quality of relationships (*n* = 8, 42%).

### Change actors’ awareness and/or knowledge of the network

Thirteen articles (24.5%) described interventions that altered networks by changing actors’ knowledge or awareness of their position in the network or potential actors to connect [62, 70, 74, 75, 78, 83, 85, 88, 90, 92–95]. These types of strategies often (*n* = 6) sought to enhance individuals’ knowledge of their own network by having them draw a personal network map and reflect on it. Others expanded individuals’ knowledge of others in the network by providing inventories, directories, or databases. To enhance actors’ awareness and recognition of others in their network with a particular role or resource, some strategies labeled an individual (e.g., as an expert or ambassador). Finally, two studies described how actors were brought together for in-person ‘get to know you’ sessions to enhance their awareness of one another. Although these might have incorporated strategies that are addressed in the “create groups” category, these interventions were designed specifically to improve actors’ knowledge and awareness about other potential connections (hence focusing on the actors rather than the group). About 85% of these studies (*n* = 11) described intended changes to tie quantity and 31% intended to change tie quality (*n* = 5).

#### Change actor prominence or role

Nine articles (17.0%) described interventions that altered networks by targeting an individual actor’s prominence, position, or role [22, 62, 78, 84, 90, 91, 95–97]. Some of these strategies combined skill or knowledge-building strategies intended to help actors enhance their own centrality in the network (e.g., to enhance actors’ ability to diffuse information, express leadership, or become less isolated within the network). Three articles described approaches where individual actors were labeled as experts or leaders to enhance their prominence in the network. Fewer (*n* = 2) described strategies for training actors to interact in different ways (e.g., not to reinforce or support certain individuals) to decentralize actors with negative behavior (e.g., bullying). Most often, these strategies intended to change tie quantity (*n* = 8, 88.9%) and few focused on tie quality (*n* = 2, 22.2%).

#### Change actors’ motivations for networking

Twelve articles (22.6%) described interventions that altered networks by changing actors’ internal motivations to interact with one another [51, 75, 78, 82, 83, 85, 90–93, 95, 96]. These strategies included efforts to educate actors about the benefits or consequences of specific types of interactions (e.g., consequences of interactions with peers who smoke on an individuals’ smoking cessation), or providing a direct incentive or reward to actors (e.g., help with household tasks) for changing their interactions in the network. Most were designed to increase interactions in the network, although some were designed to weaken or dissolve relationships. One-third (*n* = 4) targeted tie qualities, and 91.7% targeted tie quantity (*n* = 11).

#### Target and change specific ties

Fifteen articles (28.3%) described interventions that altered networks by targeting a specific tie between selected actors, or a specific type of tie for formation, strengthening or dissolution [36, 38, 54, 57, 59, 61, 64, 66, 68, 78, 88, 91, 93, 96]. Many of these approaches were used in combination with other network alteration strategies; for instance, some strategies focused on actors’ needs and characteristics to make strategic connections (e.g., matchmaking and mentorship). Others used incentives to stimulate new relationships between strategically selected groups. Nearly all sought to form or strengthen ties among actors, although some introduced educational approaches to dissolve relationships with selected actors (e.g., those with antisocial behavior). These strategies tended to target tie quantity (*n* = 13, 92.9%) although half targeted tie quality (*n* = 7, 50%).

## Discussion

Network alteration interventions are deliberate efforts to change networks by adding or removing network actors (nodes), relationships (ties), or modifying existing relationships. Given the emphasis on building strategic networks for implementation, clarifying network alteration strategies has the potential to enhance the specificity and effectiveness of various implementation strategies. Based on a sample of empirical studies of network alteration interventions in a variety of settings, we conducted an iterative synthesis. Through this process, we generated a typology of eight network alteration strategies. These strategies targeted the general context, actors, and specific ties to change the overall connectivity within networks or with focal actors. We discuss how these strategies build on earlier work on network interventions, implications for enhancing the operational specificity for implementation strategies, and areas that require additional development and research.

### Specific network alteration strategies and implications for implementation strategies

**Fig. 3:**
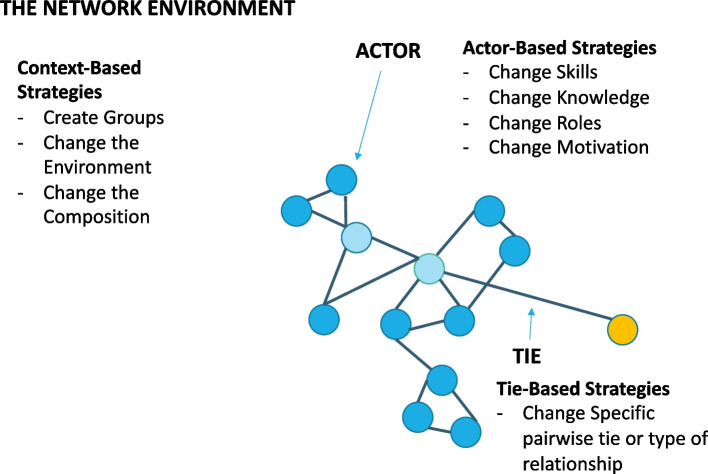
Network alteration strategies

Eight specific network alteration strategies emerged that target structural features at each level of the network. These strategies varied in their precision and focus, ranging from those that targeted the general context to those directly targeted specific features of the network (e.g., changing a particular tie) (Fig. 3). These strategies complement Valente’s earlier work [7] that proposed three general approaches for altering networks (introduce or remove actors or ties, and change existing relationships) by offering operational specificity and clarifying the multi-level targets for these interventions.

*Context-based* alteration strategies focused on the environment, culture, and resources by (1) creating groups, (2) changing the environment, or (3) changing the network composition. These strategies involved an external change (an exogenous effect) to the context of the network that modified interaction opportunities but did not try to change natural or endogenous networking tendencies. Rather, context-based strategies seemed to depend on endogenous network effects to drive changes in the number and nature of relationships. For instance, Long and colleagues described the creation of a coalition (create a group) of scholars from a similar field to increase collaboration; the coalition did not intervene with specific members or relationships, but rather created the structure and opportunity for natural endogenous networking [53].

Several implementation strategies included in the ERIC strategy taxonomy include context-based alteration strategies [5, 6]. For instance, strategies like building a coalition, creating implementation teams, and conducting a learning collaborative create new groups. These new groups could foster interactions that support shared learning and information exchange for implementation. Implementation strategies like creating a dissemination organization or increasing demand for evidence-based practice change the environment and might stimulate new relationships among organizations and practitioners in response, and support adoption and implementation. Strategies that introduce an external implementation facilitator or technical assistance provider into a clinic change the composition of clinicians’ social networks and enhance their access to specialized knowledge for implementation. For these types of implementation strategies, network changes might have a mediating effect on implementation outcomes. Awareness of how networks change when these implementation strategies are introduced could inform how they are tailored to the unique social context.

*Actor-based* strategies focus on individual actors’ knowledge, skills, motivations, and prominence. These strategies altered networks by targeting actors’ skills [4] and motivations for networking [7], knowledge of surrounding actors [5], and actors’ roles in relation to others [6]. Consistent with previous scoping reviews, actor-based strategies were common [98]. Actor-based strategies are grounded in the assumption that by changing how actors think about and approach their interactions, actors will increase, strengthen, or dissolve their relationships. For example, van Asselt-Goverts and colleagues [90] provided social skills training and coaching to individuals with mild intellectual disabilities to increase the number of their social relationships and the frequency of their interactions to improve social support and well-being. Compared to context-based alteration strategies, actor-based strategies target specific changes in the way actors think and behave in the network, reflecting a more precise intervention, although the actor ultimately has discretion over when, how, and with whom they interact. These strategies complement endogenous effects (natural networking tendencies) and could be useful in helping actors build on their existing network.

Current implementation strategies listed in taxonomies do not state an explicit focus on changing how actors think about or pursue network relationships (with an intention to alter their relationships). Yet there is potential to incorporate actor-based alteration strategies during training and education. For instance, training might target clinicians’ skills for working in interprofessional implementation teams to support the formation and maintenance of collaborative ties. Actor-based strategies might also be incorporated into other educational strategies (e.g., develop and distribute training materials, or revise professional roles) to build actors’ knowledge, skill, and motivations for shifting their relationships to support implementation.

Finally, *tie-based* strategies focus on certain relationships between particular actors or groups. Tie-based strategies altered social networks by targeting a specific pair-wise relationship (e.g., building a relationship between a mentee and mentor) or relationship type (e.g., encouraging resource sharing among pairs of existing friends). Although tie-based strategies are often accompanied by actor-based strategies that target individuals’ motivation, action, and behavior, tie-based strategies are distinct because they target a specific relational component of the network. Tie-based strategies involve precise identification of, and intervention on a specific relational element in the network. This might involve deliberately building a friendship between a child with autism spectrum disorders and a child without an autism disorder [54], or ending a friendship with a peer who could have a harmful influence on an adolescent (e.g., a smoker or bully) [81, 96]. These tie-based strategies might change or “override” endogenous networking tendencies to form or maintain relationships with known or popular partners (rather than an unfamiliar or isolated actor). In other words, they have the potential to foster relationships that might not happen on their own. Implementation strategies like developing community-academic partnerships, resource-sharing agreements, network weaving, and shadowing other experts all focus on building or changing a specific relationship within the network to achieve an implementation goal.

Of note, many interventions used multiple network alteration strategies. Considering how multifaceted strategies are often needed for complex change initiatives like implementation, this is unsurprising [5]. Using combinations of network alteration strategies might enhance the effectiveness of network change and implementation outcomes. For instance, creating groups (e.g., an implementation team) might be more effective if accompanied by changing actors’ motivations and skills for working together. Similarly, coupling strategies that change actor prominence (by designating someone as an implementation champion – which focuses on an individual actor) with those that improve other actors’ awareness of these changed roles (which focuses other actors’ knowledge) might be needed to increase the number and reciprocity of advice-seeking and discussion ties with a champion. These hypotheses require empirical testing.

Moreover, many network alteration strategies were led by a facilitator who could coordinate and support network actors. In implementation, this role might be carried out by implementation support practitioners (e.g., facilitators, implementation technical assistance providers) or champions who are responsible for building and leveraging relationships with clinicians and leaders for implementation [99, 100]. Developing manuals and training modules that explain the conditions under which these strategies could be used, and how to execute them might be important for promoting the skillful delivery of network alteration strategies for implementation in the future.

### Emphasis on building tie quantity in interpersonal social networks and implications for other strategy targets

The interventions identified in our review often targeted interpersonal relationships (e.g., discussion, information-sharing, advice-seeking, and friendship). This finding is consistent with other reviews of network analysis applications in health and implementation [37, 101]. It suggests a need for developing and testing network alteration strategies for other types of actors like organizations, coalitions, and teams. This is especially important considering how effective implementation efforts must often target relationships at multiple levels of the service delivery system [102].

Network alteration strategies also emphasized building or increasing the quantity of interpersonal social relationships. Research teams expected to observe increases in metrics like degree (the number of ties with a focal actor), or network density (the number of reported relationships divided by the number of all possible relationships, which represents overall connectivity). Fewer interventions focused on changing network sub-structures (e.g., dyadic relationships, clusters, cliques) or the quality of relationships (e.g., frequency of interaction, positive/negative social interactions exchanged, or multiplexity which is the number of ways that actors are interacting).

The lack of interventions that target network sub-structures is an important gap that deserves attention because more connectivity is not always better. Relationships take time and effort and can impose expectations that can constrain actors. Especially in larger networks where dense connections among every actor are not possible, small clusters, cliques, or dyadic relationships might be more important structural features. For instance, mental health networks organized around tightly coordinated organizational cliques generated better client outcomes than those with dense connections [103]. Among individuals, strong social support delivered via hierarchical clusters of supportive peers was associated with substance use disorder recovery [45, 104]. Even when connectivity might be beneficial for an individual (i.e., enhancing access to resources), it might not lead to better performance or outcomes for a team or group [105].

### Limitations

Our findings should be interpreted in light of several methodological considerations. First, our literature search methods and inclusion criteria might have led us to miss other types of network alteration interventions that did not cite influential papers or use our search terms. We also excluded studies that examined changes in social networks around policy or practice initiatives that were not explicitly designed to alter social networks (e.g., studies of how networks change among actors participating in group quality improvement initiates, or in response to other types of environmental changes [44, 106]. These types of “accidental” network interventions can alter networks in ways that impact implementation outcomes. However, we believe that the chances of missing additional network alteration strategies by excluding these studies were minimal because our search and screening procedures still generated a robust sample of studies with sufficient operational descriptions for deep analysis. In the future, our typology might be useful for further clarifying the specific network alteration strategies used in policy and practice initiatives and the social and psychological processes within networks that diffuse information and influence [107].

Second, our team-based typology development process might have influenced the specific strategies we identified. Given the broad and interdisciplinary interest in social networks and network interventions, we cast a wide net in our search. We used a highly iterative and intensive team-based process to calibrate our shared understanding of concepts to promote consistency in the application of our methods and interpretation of results. In fact, we focused on a subset of studies initially to familiarize ourselves with the heterogeneity in the literature [108]. While we believe this is a strength of our work, it is possible that a different team might have generated other network alteration strategies.

Third, our review does not speak to the rigor of the study designs used to examine network alteration interventions or their effectiveness. Consistent with scoping reviews, we did not conduct a quality appraisal of the literature. Investigating the rigor of study designs and methods will be an important future direction that has the potential to advance best practices for research designs that experiment with and in social networks.

### Directions for advancing implementation strategies that alter social networks

Our review and synthesis lays the foundation for future research on strategies that alter social networks for implementation. One first critical step involves further specifying and operationalizing these network alteration strategies. During our review, we observed that manuscripts varied in how the network alteration interventions were described, with limited explanation of the underlying theory of change (e.g., specific explanations about why the intervention was expected to change the network, and in turn, change the outcome of interest). This information is important for further specifying strategies, their conceptual targets, and implementation outcomes. To address this gap, studies that refine and advance theory are essential for clarifying how and why these strategies influence implementation outcomes. Borgatti and Halgin distinguish between *theories of network effects* and *theories of networks* [109] and we argue that both are needed for advancing implementation strategies that alter networks. Theories that explain *network effects* (how and why networks influence implementation outcomes) are necessary for identifying the specific types of structural features that should be targeted by network alteration strategies during implementation. For example, theories about weak ties, diffusion of innovation, and bridging capital are useful for explaining how new ties between agency leaders in two different systems might lead them to learn about evidence-based interventions and adopt them. Theories about strong ties, clustering, and bonding social capital are useful for explaining how building dense relationships among clinicians might be important for improving support for implementation [110]. Studies that identify network features associated with implementation outcomes will be important for refining theories of network effects.

At the same time, we also need strong *theories of networks* that explain how and why network structures change. Although some theories of networks have been developed in disciplines like communications [111], or organizational science [26], these theories have received far less attention than theories of network effects [109]. These network theories could be used to further specify the network alteration strategies identified in this work, and tested in implementation. Importantly, connecting theories of network change and effect is necessary to clarify mechanisms of action underlying implementation strategies that alter social networks. Ultimately, these insights have direct implications for informing strategy selection during implementation. For instance, a facilitator might use a tie-based strategy to build weak ties between two leaders to help them learn about (and adopt) an evidence-based intervention. Alternatively, a clinic administrator might create an implementation team to build strong ties among clinicians to support fidelity.

A second step involves an in-depth review of existing taxonomies of implementation strategies (e.g., ERIC), to examine which strategies incorporate network alteration approaches. Bartley, Metz, and Fleming [4] recently examined ERIC strategies from a relational perspective and applied structured definitions of relational and transactional features. A similarly structured and systematic approach would be useful to identify potential incorporation of network alteration strategies in existing ERIC categories, or the need to expand existing taxonomies. Overlaying these results with those from the Bartley, Metz, and Fleming [4] study would also be useful for specifying the nature and purpose of relationships, and their mechanisms of action.

Third, introducing a network analysis lens to implementation strategies by advancing the proposed typology will also help sensitize implementation strategies towards equity-focused implementation. Analysis of social network structures can reveal power dynamics and social hierarchies at the interpersonal, organization, and structural level. Network alteration interventions have potential to connect diverse actors and social clusters to improve access, connectivity, and upward social mobility with the goal of challenging structural inequity. For instance, a tie-based strategy might be used to deliberately connect isolated actor to promote inclusion, or to create clusters of actors with similar backgrounds and characteristics for peer support and empowerment. Alternatively, actor-based strategies could be customized for different actors based on their existing skills, motivations, and local network compositions; an example might involve training primary care clinics to collaborate with culturally specific service organizations to improve healthcare access. Considering how individual actors might respond differently to group creation and context-based strategies depending on their positions in the social networks (e.g., actors at the center of a network might respond, whereas those on the periphery might not), these strategies might require tailoring and engagement of diverse actors and social groups. The proposed typology should be assessed empirically in implementation studies, to examine the effectiveness of these strategies for promoting equity in reach, access, engagement, effectiveness, and sustainment, and their ultimate impact in equitable implementation. This further highlights the importance of mixed methods network analysis approaches [112–114].

Fourth, each alteration strategy should be subjected to rigorous empirical testing to determine the distinct impact on network structure, and implementation outcomes. Considering how many interventions in this review used multiple strategies (e.g., interventions that created groups and increased actors’ knowledge of the network), it is also important to understand how strategies are combined, whether they achieve similar or complementary purposes, and their additive impact. The timeframe and dose needed to produce network changes are also unclear. Because network alteration strategies, (like implementation strategies) are often bundled, it can be difficult to understand the unique contribution of each strategy. Complex adaptive designs like sequential multiple assignment randomized trials might be useful for examining the unique and interacting effects of each strategy. Evidence and insights about the theoretical mechanisms of action, and effectiveness of network alteration strategies will be important for improving their precision for targeting specific features of the environment, actors, and their relationships during implementation.

## Conclusion

Many implementation strategies included in the ERIC taxonomy focus on building social networks but lack clarity and specificity. Network alteration interventions have the potential to further enhance implementation strategies theoretically and specify them practically. Through our review and synthesis, we identified eight network alteration interventions. Three strategies targeted the general context: (1) change the environment, (2) create groups, and (3) change the composition. Four strategies targeted individual actors: change (4) motivations, (5) skills for networking, (6) knowledge of one’s social network, and (7) prominence/roles. One strategy (8) targeted specific ties. Future research is needed to further specify, test, and adapt these strategies for changing network structure for implementation.

## Supplementary Information

**Additional file 1.** Stage 2 Codebook.

## Data Availability

The data generated and analyzed for this study are available at https://airtable.com/shrqMjdUzMsixxx68.

## Abbreviations

ERIC: Expert Recommendations for Implementing Change
PRISMA: Preferred Reporting Items for Systematic Reviews and Meta-Analyses
