# Supplementary material for: Developing a typology of network alteration strategies for implementation: a scoping review and iterative synthesis
Source: Implement Sci. 2023 Apr 6;18:10. doi: 10.1186/s13012-023-01266-3 (PMC10080780; doi:10.1186/s13012-023-01266-3)
Supplement: Supplementary file 1 — Additional file 1. Stage 2 Codebook. [file 13012_2023_1266_MOESM1_ESM.docx]

**Supplemental File 1: Stage 2 Codebook**

| **Field** | **Codes** | **Definition** |
| --- | --- | --- |
| Coder Initial | -- | Initials of the team member |
| Article ID | -- | Internal identification number for article |
| Article First Author | -- | Last name of the article’s first author |
| *Nodes/Actors* | | The type of nodes/actors that were in the network. |
| Individuals | Y/N | Nodes represented individual people |
| Small group | Y/N | Nodes represented a small group like a team, group, class. |
| Organizations | Y/N | Nodes represented a formal organization |
| Coalitions | Y/N | Nodes represented formal or informal coalitions |
| *Intended Network Change* | | Features of the network that are expected to change |
| Tie Quality | Y/N | Whether the intervention intended to change the quality of the ties in the network. This includes changing the frequency of interactions, the nature or content of the relationship, or the influence of the ties. |
| Tie Quantity | Y/N | Whether the intervention changed the quantity of ties within the network. |
| *Level of Intervention* | | The network components targeted reflected by metrics |
| Whole network | Y/N | Structural metrics that reflect properties of the entire network (e.g. density, centralization) |
| Sub-groups | Y/N | Structural metrics that reflect properties of network subgroups like cliques or clusters. |
| Dyads | Y/N | Structural metrics that reflect properties of dyadic (pair-wise) relationships like the presence/absence of a tie, or reciprocity. |
| Triads | Y/N | Structural metrics that reflect properties of triadic (three way) relationships like transitivity. |
| Individual | Y/N | Structural metrics that reflect properties of individual nodes/actors based on their networking activity like individual degree or centrality. |
| Network boundary | Y/N | Metrics or properties that reflect network boundaries like size. |
| *Network Alteration Strategies* | | The strategies, methods, or approaches used to change network structures (the “how”). |
| Create Groups | Y/N | Whether the intervention altered networks by creating groups (e.g., bring actors into closer physical, virtual, social proximity). |
|  | Type | One sentence description of the types or nature of the groups created. |
|  | Strategy | One sentence describing strategies or approaches to creating groups. |
| Change the environment | Y/N | Whether the intervention altered networks by creating new environmental conditions like funding, regulations, shock/disaster. |
|  | Domain | One sentence description of the aspect or domain of the environment that was changed. |
|  | Origin | One sentence description of who or what was in control of the environmental changes (the change origins) |
| Change the composition | Y/N | Whether the intervention altered networks by changing who or what is in the network |
|  | How | Whether the composition is changed by adding or deleting nodes. |
| Change actor skills | Y/N | Whether the intervention altered networks by changing actors’ skills |
|  | Type | The type of skills targeted by the intervention |
|  | Component | The intervention components that targeted actor skills (e.g. workshops, trainings, role-play) |
| Change actor knowledge | Y/N | Whether the intervention altered networks by changing actors’ knowledge or awareness |
|  | Component | The intervention components that targeted actors’ knowledge (e.g. training, developing personal network maps) |
| Change an actor’s prominence, position, role. | Y/N | Whether the intervention altered networks by changing an actor’s individual prominence, position, or role. |
|  | Approach | A one sentence description of how the intervention changed an actor’s role. |
| Change actors’ incentives or motivations | Y/N | Whether the intervention altered networks by changing incentives or motivations for an actor to network |
|  | Actor type | The types of actors targeted by the intervention |
|  | Mechanism | One sentence description of how the intervention changed incentives or motivations. |
| Change ties | Y/N | Whether the intervention altered networks by changing specific ties among actors (e.g. Brokering or breaking up ties) |
|  | Tie Type | Specific types of relationships that were targeted (e.g., friendships, connections between specific types of actors) |
|  | Goal | Whether the intervention intended to form, maintain, dissolve, or change the nature (e.g., type, frequency) of the tie. |
